# Supplementary material for: Impact of rural soundscape on environmental restoration: An empirical study based on the Taohuayuan Scenic Area in Changde, China
Source: PLoS One. 2024 Mar 18;19(3):e0300328. doi: 10.1371/journal.pone.0300328 (PMC10947686; doi:10.1371/journal.pone.0300328)
Supplement: Attachment — Submitted filename: Response to Reviewers.docx [file pone.0300328.s002.docx]

Dear Editors and Reviewers:

We would like to sincerely thank for your help and thank for reviewers’ professional comments concerning our manuscript entitled “A study on the mechanism of the impact of rural soundscape perception on environmental restoration: An empirical study based on the 3

Taohuayuan Scenic Area in Changde, China”(Number:PONE-D-23-26044). Those comments are all valuable and very helpful for revising and improving our paper, as well as the important guiding significance to our research. We have revised the manuscript carefully according to the comments and suggestions of reviewers and editors and responded point by point to the comments as listed below. The revised manuscript has been edited and the revised part are highlighted in red.

**Responds to the editor’s comments:**

1. **Please ensure that your manuscript meets PLOS ONE's style requirements, including those for file naming.**

Thanks very much for your comments.We have revised our manuscript based on PLOS ONE's style template and hope to meet the journal's stylistic requirements.

2. **You indicated that ethical approval was not necessary for your study. We understand that the framework for ethical oversight requirements for studies of this type may differ depending on the setting and we would appreciate some further clarification regarding your research. Could you please provide further details on why your study is exempt from the need for approval and confirmation from your institutional review board or research ethics committee (e.g., in the form of a letter or email correspondence) that ethics review was not necessary for this study? Please include a copy of the correspondence as an ""Other"" file.**

We are grateful for the suggestion.By carefully reading the journal's requirements for ethical approval again, this study did involve Human participants, so ethical approval was applied for and relevant supporting materials were uploaded into the system.

3. **In your Data Availability statement, you have not specified where the minimal data set underlying the results described in your manuscript can be found. PLOS defines a study's minimal data set as the underlying data used to reach the conclusions drawn in the manuscript and any additional data required to replicate the reported study findings in their entirety. All PLOS journals require that the minimal data set be made fully available.**

We are sorry for this problem and have uploaded our minimal data set as a Supporting Information file to the system under the file name paper data.

4. **PLOS requires an ORCID iD for the corresponding author in Editorial Manager on papers submitted after December 6th, 2016. Please ensure that you have an ORCID iD and that it is validated in Editorial Manager.**

In accordance with the requirements of the journal, the corresponding author of this study have made available in the Editorial Manager the ORCID iD.

5. **We note that Figure 2 in your submission contain map images which may be copyrighted. All PLOS content is published under the Creative Commons Attribution License (CC BY 4.0), which means that the manuscript, images, and Supporting Information files will be freely available online, and any third party is permitted to access, download, copy, distribute, and use these materials in any way, even commercially, with proper attribution. For these reasons, we cannot publish previously copyrighted maps or satellite images created using proprietary data, such as Google software (Google Maps, Street View, and Earth).**

We really appreciate editor's valuable comment for this point.The maker of Figure 2 is one of the authors of this study and we have submitted the original copyright holder's Content Permission Form as well as written permission in the system.

6. **Please ensure that you refer to Figure 2 to 4 in your text as, if accepted, production will need this reference to link the reader to the figure.**

We are grateful for this suggestion.We have revised the issue and have ensured that Figures 2 to 4 are mentioned in the paper.

7. **We note you have included a table to which you do not refer in the text of your manuscript. Please ensure that you refer to Table 1 in your text; if accepted, production will need this reference to link the reader to the Table.**

We are grateful for this suggestion.We have revised the issue and have ensured that the paper refers to Table 1.

**Responds to the reviewer’s comments:**

**Response to Reviewer #1**

1. **Abstract：please include who are the respondents of the study.**

Thank you for your valuable suggestions to improve the accessibility of our manuscript. We have added the respondents of the study to the abstract. We have copied the selection of abstracts below the response and **the requested changes are presented in bold font（Line 18, page 1）** for your quick reference.

Previous studies on environmental restorative effects have mainly fo-cused on visual landscapes, and less on the influence of soundscapes on restora-tive, but soundscapes play a crucial role in restorative environments, especially rural soundscapes, but there is insufficient existing theoretical evidence on the subject. Therefore, this study aims to investigate the influence of Rural Sound-scape Perception on Environmental Restoration Perception, and introduces two affective variables, tourism nostalgia and place attachment, to explore the mechanism of Rural Soundscape Perception on Environmental Restoration Per-ception, as well as the moderating role of the number of trips is also discussed. Based on the theory of restorative environment, this study took the Taohuayuan Scenic Spot in Changde, Hunan Province, China, as the case site, and **selected the rural soundscape in the area as the research object;** a total of 506 valid data were collected through questionnaire surveys, and structural equation modeling was used to validate the collected data.

2. **Introduction：this section should contain the knowledge gap, and to explain why it is important to address the gaps in knowledge.**
We are very grateful to reviewer #1 for his/her precious feedback on our paper. Based on the reviewer's valuable comments, we have refined the introduction to further clarify knowledge gap and the importance of addressing it, and the revisions are organized as follows:

**Firstly,** **from the rural landscape.** Early tourism scholars due to the "tyranny of vision" and its soundscape and other sensory aspects of the research is not yet in place, the research on rural tourism landscape is more concerned about the design and improvement of the visual landscape, ignoring the auditory perception of the construction of the soundscape and its implied value analysis. However, while visual perception is important, the sound landscape represented by auditory perception should not be neglected. Sound is a key link in enriching and enriching tourists' local perception and experience, and it not only affects tourists' experience through physical properties such as sound level, but also has cultural and social existence. Many studies have confirmed the importance of rural soundscape in rural tourism destinations, and how to optimize and create a satisfactory rural soundscape for tourists has become an urgent problem for scholars.**（Line 61-78, page 2）**

**Secondly**, from environmental restorative theory. Early research on environmental restorative theory focused on visual perception restoration. However, the role of soundscape in enhancing environmental restorative effects is equally not negligible. Compared with visual scenes, sound has a more significant effect on people's recovery from fatigue and worry in some situations. With the rise of the soundscape research field, more and more scholars have begun to pay attention to the restorative effects of auditory stimuli, and have gradually confirmed the health restorative effects of soundscape in physiological and psychological aspects, but the relevant theoretical evidence has yet to be improved.**（Line 127-148, page 3）**

**Thirdly**, from the restorative effects of rural soundscapes. It has been shown that countryside soundscapes can have a restorative effect on human psychology and physiology. Meanwhile, among the scenes of urban soundscape, urban park soundscape and rural soundscape, rural soundscape has the highest restorative potential, which shows that the restorative effect of rural soundscape is of great research value. However, previous studies have mainly focused on the restorative effects of soundscapes in urban spaces, such as city parks, forest parks, community parks and campuses, and fewer scholars have studied the restorative effects of countryside soundscapes on the environment, while countryside tourist destinations, as an important place for city dwellers to alleviate their stress and relax their moods, take on the role of providing restorative environments, whose restorative research deserves to be paid attention to.**（Line 148-166, page 4）**

**Finally**, from the mechanism of rural soundscape perception on environmental restorative perception. Although a certain amount of research has been carried out on the restorative effect of rural soundscape in the past, the mechanism of rural soundscape on the restorative effect of the environment is still unclear, and the guiding significance of rural landscape planning is limited. Therefore, it is necessary to study in depth the influence mechanism of rural soundscape on environmental restorative effect. In this study, two emotional factors, tourism nostalgia and local attachment, are introduced into the mechanism.**（Line 167-172, page 4）**

3. **The literature review requires updating as majority of them are old.**
We thank for Reviewer #1 for highlighting this point. We have updated the literature in the Literature Review section, but we are unable to present the references in its entirety to you because we have combined the Introduction and References sections based on the comments of another reviewer. However, we have updated the literature by counting the number of references, and we hope to present them in a numerical way so that you can better observe the results of the revision. **According to the statistics, there are 4 literature updates in 2019; 4 literature updates in 2020; 5 literature updates in 2021; 7 literature updates in 2022; and 3 literature updates in 2023, totaling 23 updates.**

**References:**

1. Liu P.P. A Study on the Soundscape Design of Zhujiayu Village Based on Homesickness Memory. Thesis, Shandong University, 2020.
2. Huang, W. Research on Rural Soundscape in the Context of Rural Tourism in Southern Hebei. Thesis, Hebei University of Engineering, 2023.
3. Rao Y.Y.; Wu X.H.; Liang M.X.; Wu Y.X. Study on Soundscape Evaluation and Its Influencing Factors of Longjing Village in Hangzhou. Noise Vib. Control 2021, 41, 212–218.
4. Chen M.C.; Qu G.C. Soundscape Evaluation in Rural Tourism Destinations: A Case Study of Hongcun Village in Southern Anhui Province. J. Hebei Univ. Environ. Eng. 2021, 31, 52–55, doi:10.13358/j.issn.2096-9309.2020.1206.03.
5. Wang C.Y.; Huang W.; Feng H. Evaluation study on soundscape of rural tourist sites in Ji’nan area. Tour. Overv. 2022, 25–28.
6. Li, S.H.; Wang, C.S. Study on Planned Preventive Conservation of Tourism Traditional Villages: Taking Shifeng Village of Chaozhou City as an Example. Urban Dev. Stud. 2021, 28, 10-15+21.
7. Zeng J. Study on the perception evaluation of rural soundscape in Guanzhong area. Thesis, Xi’an University of Architecture and Technology, 2020.
8. Jin, Z. Research on the current situation of soundscape of traditional villages in Huizhou——Taking Xidi Town, Yixian County, Anhui Province as an example. Hous. Ind. 2023, 40–43.
9. Han F.Q. Evaluation, Protection, and Utilization of soundscape in Traditional Villages of Dong Nationality - Take Zhaoxing Dong Village as an Example. Thesis, Hebei University of Engineering: Hebei, 2023.
10. Jiang Z.W. Research on Grey Space Design of Meishan Cultural Park Based on Symbiosis Theory. Thesis, Hunan University: Changsha, 2022.
11. Wang, J.F. Research on the Rural Tourism Experience Based on Soundscape Perception. Thesis, Hunan Normal University: Changsha, 2019.
12. Wang, S.Y.; Huang, W.; Feng, H. A Study on the Relationship Between Rural Soundscape Perception and Tourists’ Willingness to Behave. J. Hebei Univ. Environ. Eng. 2022, 32, 10–15, doi:10.13358/j.issn.2096-9309.2022.0512.02.
13. Liu, C.; Li, S.H. Review of Restorative Natural Environment under Multidisciplinary Perspectives. Chin. Landsc. Archit. 2020, 36, 55–59, doi:10.19775/j.cla.2020.01.0055.
14. Cui, H.L. Study on the influence of wetland tourists’ environmental restoration perception and satisfaction on revisit intention. Thesis, Hubei University: Wuhan, 2021.
15. Kjellgren, A.; Buhrkall, H. A Comparison of the Restorative Effect of a Natural Environment with That of a Simulated Natural Environment. J. Environ. Psychol. 2010, 30, 464–472, doi:10.1016/j.jenvp.2010.01.011.
16. Wang, X.X.; Wu, C.Z.; Yan, J. Experimental Study of the Perceived Restorative Scale (PRS) in Chinese by Evaluating the Restorative Qualities of Urban Park Scenes. Chin. Landsc. Archit. 2019, 35, 45–48
17. Fu E.K.; Wang Y.H.; Zhou J.W.; Li X. A Tentative Research of Restorative Environmental Evaluation of Community Parks Base on Eye Movement Analysis. South Archit. 2022, 93–99.
18. Chen, H.L.; Zhang, L.J.; Zhao, Z. Effect of Tourists’ Perceived Restorative Environment on Place Attachment and Loyalty—A Case of Bama Yao Ethnic Group Autonomous Couty in Guangxi. Resour. Dev. Mark. 2021, 37, 1239–1246.
19. Li, J., Zhongzhe Kang Sensitivity Analysis of Changes in Human Physiological Indicators Observed in Soundscapes. Landsc. Urban Plan. 2019, 190.
20. Zhao J.W.; Xia T.T. Effects of Soundscape on Mental Restoration in Urban Green Space. Landsc. Archit. 2019, 26, 83–88, doi:10.14085/j.fjyl.2019.05.0083.06.
21. Hu G.M.; Li F.Y. Progress in empirical research on restorative effects of soundscape in urban open spaces. Sichuan Archit. 2021, 41, 55–58.
22. Wen, P. Research on the Development of Rural Health Tourism from the Perspective of Restorative Environment. Agric. Econ. 2022, 98–100.
23. Zhang, H.B.; Chen, G.S.; Liao, F.; Chen, H.Q.; Yu, K. Construction of Evaluation Index System of Acoustic Landscape in Rural Tourist Attractions and Its Optimal Design Path. J. Chang. Univ. 2021, 35, 67–76.
24. Szkopiecka, A.; Wyrwa, J.P.; Chrobak, G.; Kołodyńska, I.; Szewrański, S. Perceived Restorative Potential of Urban Parks by Citizens—A Case Study from Wrocław, Poland. Sustainability 2023, 15, 7912, doi:10.3390/su15107912.
25. Guo, Y.; Jiang, X.; Zhang, L.; Zhang, H.; Jiang, Z. Effects of Sound Source Landscape in Urban Forest Park on Alleviating Mental Stress of Visitors: Evidence from Huolu Mountain Forest Park, Guangzhou. Sustainability 2022, 14, 15125, doi:10.3390/su142215125

4. **In the discussion section, the authors could add further explanation on the insignificant moderation relationship.**

Special thanks to Reviewer #1 for his/her comments. We have further explained the insignificant moderating relationship in the Discussion section. It reads as follows:

**About H3a：historical nostalgia significantly and positively affects Environmental Restoration Perception, while personal nostalgia does not have a significant effect on Environmental Restoration Perception.**

**This result can be explained by several reasons. Firstly，This research reflects on the definition of tourism nostalgia:** personal nostalgia consists mainly of events actually experienced by individuals in the past. Some researchers have argued that nostalgia is a negative emotion involving sadness and pain in an individual's past. It is the process of people's remembrance of the good things in the past, and when the good things that have passed away no longer exist or cannot be reproduced, people feel sadness and helplessness, and this nostalgic emotion of feeling deep remembrance for the things that have passed away is more likely to be the motivation for individuals to escape from the daily routine and to seek after the other, and it becomes an effective way for people to escape from modernity . Therefore, personal nostalgia may be more closely related to tourism nostalgia and have a less significant effect on Environmental Restoration Perception, which has been confirmed by previous scholars' studies .Historical nostalgia focuses on the scenes of people's lives in the past, which can be events that happened in history or memories of a specific historical era . Nostalgia has an aesthetic character, and the subject of nostalgia tends to desire and pursue beautiful things. Since historical nostalgia is an individual's imagination of the past that he or she has not experienced, there is a large space for imagination, therefore, people tend to miss the beautiful and happy historical memories, which is a kind of positive nostalgia emotion that helps to alleviate people's mental stress and physical and mental exhaustion, and then promotes restorative nature. **Secondly, thinking from the perspective of social phenomenon:** the content of personal nostalgia requires real experience, so it is highly subjective, i.e., different experiences of individuals lead to different nostalgia produced by individuals . With the acceleration of urbanization, more and more people are moving from the countryside to the cities, and China's urbanization rate has reached 64.72% as of 2021. The accelerated urbanization, on the one hand, makes many people unfamiliar with the countryside and its soundscape, especially contemporary children or young people, because most of them grew up in the city and know more about the countryside from electronic devices and books, and thus may have a focused and biased memory of the countryside. Therefore, then, the lack of authentic countryside experiences leads to unfamiliarity with the countryside, which in turn makes it difficult for tourists to develop personal nostalgia during tourism. It has been confirmed that the more familiar an individual is with the environment he or she is in, the stronger his or her Environmental Restoration Perception is. So when the familiarity of a place is not enough for tourists to produce personal nostalgia, the lower their Environmental Restoration Perception, so the effect of personal nostalgia on Environmental Restoration Perception is not significant. On the other hand, the acceleration of urbanization has also changed the appearance of the traditional countryside, making the artificial and mechanical sounds in the countryside increase, which makes the tranquil countryside soundscape different from the countryside soundscape in people's recollections, which leads to feelings of sadness and helplessness, and this negative emotion is not conducive to promoting restorativeness.**（Line 760-803, page 23-24）**

**About H10：the number of trips does not moderate the relationship between perceived rural soundscape and environmental restoration perception.**

It is suggested that, in the context of rural soundscape perception, the number of trips does not influence environmental restoration perception. This finding is similar to Guo et al.'s (2014) study, which found that the number of trips does not have a significant impact on tourists' perception of environmental restoration. It is also important to note that not all soundscapes in rural environments are comforting sounds, and certain rural tourist locations may be filled with a large amount of uncomfortable noise, such as mechanical sounds. Therefore, it is the environment in which the traveler is located that should influence individual restorative effects, not the number of trips, and thus the number of rural travel experiences may not play a moderating role in the structural relationship between Rural Soundscape Perception and Environmental Restoration Perception. It has been found that the length of travel time has a significant effect on the effect of restorative environmental perception, and the longer the stay, the higher the restorative experience, as well as the factors of place memory, place dependence and place identity, and familiarity of the environment positively affect people's Environmental Restoration Perception, so it can be seen that tourists' Environmental Restoration Perception may be more related to the length of tourists' travel time , familiarity of the environment, local memory and other factors, while it is not closely related to the number of tours.**（Line 838-852, page 25）**

5. **The conclusion section should include discussion that are connected to the empirical results to the theoretical as well as literature.**

We are grateful for this suggestion. We have added some discussion of the empirical results and the theory and literature associated with them in the conclusion section, but probably not much. This is because we have centralized the discussion of empirical results and theory and related literature in the discussion section.Additions are bolded in black below**（Line 865-870, page 25）.**

This study explores the environmental restorative effect of tourists based on the perspective of soundscape perception and introduces the relation-ship of a total of four variables, namely, rural soundscape perception, tourism nostalgia, place attachment, and environmental restoration perception, to rural tourist places for empirical research, and constructs a mechanism for the influence of rural soundscape perception on environmental restoration perception. At the same time, it also reveals the complex path of "rural soundscape perception→tourism nostalgia→place attachment→environmental restoration perception", which provides a new perspective for the understanding of the mechanism of the rural environment and people's health, and to a certain extent, broadens the research of the combination of soundscape ecology, landscape design, tourism, and environmental psychology. **Although research on the restorative effects of soundscapes has been widely used in previous restorative environmental research, there have been fewer studies on the mechanisms by which soundscapes influence the restorative effects of the environment, and insufficient emphasis has been placed on the restorative aspects of rural soundscapes.**

**6. Implication section should include practical/managerial implications as well as recommendations to the stakeholders/managers/tourism agencies.**

Thank you for your valuable suggestions to improve the accessibility of our manuscript. We've added content from the 7.1 implications section. The relevant content is provided below for your quick reference**(Line 881-926, page 26)**.

The findings of this paper show that the soundscape environment of rural tourist sites has a positive impact on tourists' restorative properties. Therefore, scenic area managers can enhance the environmental restorative effect of tourists as well as the quality of tourism experience through the optimization of scenic soundscape design. Based on this, the following suggestions for optimizing the soundscape environment of rural tourism destinations are proposed for reference and reference.

(1) Focus on tourists' soundscape expectations and design positive factors. The rural soundscape environment is a comprehensive environment that contains natural soundscape and rural cultural soundscape. The natural soundscape includes the sounds of birdsong, running water and wind blowing leaves around the village, which help people relax and feel the rural life. The rural cultural soundscape includes traditional folk music, instrumental music, dance performances and local dialects, which let people feel the charm of traditional culture and enhance the sense of cultural identity. In the rural soundscape design, we need to study and understand the characteristics of the natural soundscape and rural cultural soundscape, and reasonably integrate them into the design to provide a better experience for the villagers and tourists, so that the soundscape design can enhance the natural atmosphere and cultural heritage of the rural environment, so that people can better feel the joy of rural life, and thus can promote the environmental restorative effect.

(2) Optimize the configuration of audiovisual elements and control negative factors. Rural tourists favor quiet, tranquil atmosphere and characteristic activities, but due to large-scale tourism development and tourist crowding, traditional villages become noisy. Valuable soundscape resources such as natural sounds, sounds of folk activities and farming sounds have not been fully utilized. Therefore, the government should plan and manage beautiful soundscapes in the rural environment. While using positive factors to shape the traditional village environment, it should also try to avoid negative factors. For example, unnecessary and incongruous sounds can be controlled and reduced by planting hedges and installing sound barriers that can shield road traffic noise and mechanical noise. At the same time, some natural sounds can be created to mask the noise.

(3) Protect the traditional soundscape of the countryside and inherit the cultural factors. Tourists have a high preference for rural natural soundscape and rural cultural soundscape, and rural soundscape perception has a positive effect on environmental restorative perception. Therefore, increasing and highlighting the rural natural soundscape and cultural soundscape can influence the restorative perception of tourists to a certain extent and improve their traveling experience. Scenic area managers should first carry out natural ecological environment protection, so as to protect the rich and diverse natural soundscape in the rural scenic area. Secondly, the soundscape with rural cultural characteristics in the scenic area should be publicized and protected, such as: playing natural pure music, local representative music, etc. to reduce the noise impact inside the scenic area; in addition, through stage performances, film and television creations and other ways, the soundscape with local rural characteristics will be disseminated and diffused, so as to achieve the effect of inheritance and protection.

**Response to Reviewer #2**

**（1）Major comments**

**1. It is suggested to add key words in the manuscript.**

We are grateful for this suggestion. Keywords have been added to our manuscript.

**Keywords: rural soundscape perception; environmental restorative perception; place attachment; tourist nostalgia; rural tourism (Line 33-34, page 1);**

2. **It is suggested to improve the framework, especially the background, literature review, methods and materials.**

Thank you for your valuable suggestions to improve the accessibility of our manuscript.We have improved the framework based on your comments, especially the three sections you specifically mentioned (background, literature review, methods and materials), as described below:

**For the background: we reorganized the logic of the article**

**①First of all,** this study points out that rural tourism is favored by people's outbound travel through the problems brought by the accelerated urbanization (such as noise, environmental degradation, life pressure, etc.). At the same time, it compares the significant difference between urban sound and rural sound to **lead to the object of this paper - rural soundscape.**

**②Secondly,** a literature review of rural soundscape is conducted, and it is found that the current research on rural soundscape is deficient in two aspects, on the one hand, rural soundscape, as an important part of rural tourism, has not received enough attention, and on the other hand, there is a lack of research on the restorative aspects of rural soundscape. However, the rich sound resources in the countryside can not only create a good tour experience for tourists, but also play an important role in promoting the health of urban residents. **It further points out the importance of studying rural soundscape.**

**③Thirdly, the focus of the article, the environmental restorative effects of rural soundscapes, begins to be discussed.** Through a literature review of environmental restorative research, it is found that in terms of the senses, previous research on restorative mainly focuses on visual landscapes, thus ignoring the restorative value of soundscapes. Meanwhile, from the perspective of the research area of soundscape restorative, previous studies mainly focused on urban public space, and relatively few restorative studies involved rural areas, while rural tourist places, as an important place for urban residents to relieve pressure and relax, assume the role of providing restorative environments, whose restorative research deserves to be paid attention to. **Thus, the importance of the restorative effect of rural soundscape is further elaborated.**

**④Finally,** it is suggested that the mechanism of the influence of rural soundscape on the restorative effect of the environment is still unclear and needs to be verified, thus **presenting the main research purpose of this paper - the mechanism of the influence of rural soundscape perception on the restorative perception of the environment.**

**For the literature review:**we integrated the literature review with the introduction, thus deleting the literature review section. In the introduction section, we focused on previous research on rural soundscapes, environmental restorativeness, and the restorative effects of rural soundscapes.

**For Materials and Methods:** we also made some refinements and modifications to this section, such as moving the content on scale selection from the original literature review to the Materials and Methods section.

3. **The writing is not concise enough, and the content is slightly lengthy, while it dilutes the core content. It is suggested to simplify the manuscript.**

We really appreciate Reviewer #2's valuable comments and we have simplified all sections accordingly.

4. **It is suggested to further clarify the research questions.**

We sincerely appreciate Reviewer #2's suggestion for further clarification of the research questions. We have further clarified and refined the research questions in the introductory section as follows:

in this study, the Taohuayuan Scenic Spot in Changde City, Hunan Province was selected as a case study for empirical analysis, with the main purpose of exploring the influence mechanism of Rural Soundscape Perception on Environmental Restoration Perception by constructing a structural equation model, and introducing the two affective variables of tourism nostalgia and place attachment, to provide new research perspectives for the theoretical study of Rural Soundscape Perception, and at the same time, to provide theoretical basis for the design and creation of soundscape in rural tourism destinations and decision-making support. In addition, the reasons for the restorative effect of rural soundscape are still unclear in the academic world, and the introduc-tion of the relationship between the two variables into the rural sound envi-ronment for restorative empirical research is a novel and meaningful research topic in itself. **This study attempts to determine the impact of Rural Sound-scape Perception on Environmental Restoration Perception by answering three research questions: (1) whether Rural Soundscape Perception has an impact on Environmental Restoration Perception; (2) what are the pathways and mecha-nisms of action of Rural Soundscape Perception on Environmental Restoration Perception; and (3) whether tourism nostalgia and place attachment mediate the effect.(Line 214-232, page 5)**.

5. **In the Abstract, there is no logical connection between the first and the second sentence.**

We thank for Reviewer #2 for highlighting this point,and we reworked the first and second sentences of the abstract to make them more coherent.**（Line 8-20, page1）**

6. **From Line 44 to Line 47, I don’t think “rural soundscape perception” is a “creative concept” in this manuscript. There are some researchers who have studied rural soundscape, in addition, the concept of soundscape has been proposed in the ISO standard. This paper is not conceptually innovative. The research content and research scope are also under the scope of soundscape. It is suggested to modify.**

We really appreciate Reviewer #2's valuable comment for this point，and we have made appropriate revisions based on the reviewers' comments. **The content is shown below：**

Based on previous research, this study further rationalizes and integrates the concept of "Rural Soundscape Perception", which is defined as the perception and understanding of the surrounding sound environment by tourists during rural tourism.**（Line 90-93, page2）**

7. **From Line 74 to Line 75, what is the mean of “environmental restorative effects” referred to? In the introduction, it is not explicitly illustrated.**

We are sorry for this problem,and we have clearly explained the concept of "environmental restorative effects" in the paper.**The content is shown below：**

Environmental restorative effects refer to the positive outcomes that may occur when a person is in a natural environment characterized by recovery-promoting, stress-reducing, and restorative features.**（Line 106-108, page3）**

8. **From Line 102 to Line 116, it is recommended that the research questions would be clearly presented in this paragraph, and generally speaking, there are usually three research questions.**

We sincerely appreciate the suggestions made by Reviewer #2 regarding the research questions.We have clarified and refined the research questions based on the reviewers' comments and summarized three research questions according to the content of the paper.**The content is shown below：**

This study attempts to determine the impact of Rural Sound-scape Perception on Environmental Restoration Perception by answering three research questions: (1) whether Rural Soundscape Perception has an impact on Environmental Restoration Perception; (2) what are the pathways and mecha-nisms of action of Rural Soundscape Perception on Environmental Restoration Perception; and (3) whether tourism nostalgia and place attachment mediate the effect.**(Line 226-232, page 5)**

9. **In terms of “Literature Review”, first of all, it is suggested a combination of literature review and introduction. Secondly, the topic of the literature review is closely related to the research questions. Thirdly, texts which are not closely related to the topic can be deleted.**

By following the reviewer’s suggestion,we merged the references and introduction sections. Second, the research literature on topics closely related to the research questions (e.g., rural soundscapes, environmental restorability, and restoration effects of rural soundscapes) was organized in detail.

**( page 1-5)**

10. **In paragraph of 2.3 Environmental Restoration Perception, it is suggested to clarify the aim of this paragraph, and separated method description from literature review. Paragraph of 2.4 and 2.5 should be also changed in this way.**

By following the reviewer’s suggestion,we merged the references and introduction sections,and we have separated method description from literature review to the section of materials and methods.

**(Line 467-499, page 12)**

**11. From Line 245 to Line 246, four dimensions of “away, extensibility, charisma, and compatibility,” are inconsistent with the words in the following methods and tables.**

We thank the reviewer for bringing this to our attention. In this paper, we have combined the research review with the introduction, so the problems that existed with form Line 245 to Line 246 have been resolved, and we have checked the full paper to ensure that the four dimensions of the Revised Perceived Restorativeness Scale (distance, charisma, ductility, and compatibility) remain consistent.

**12. In paragraph of 3.1 Rural Soundscape Perception and Environmental Restoration Perception, there were two topics as restoration effects of rural soundscape and differences from Chinese rural soundscape and other research. It is suggested to focus on one topic in one paragraph.**

We are grateful for this suggestion，and paragraph 3.1 has been revised: we have moved the study of the differences between Chinese rural soundscapes and Western rural soundscapes to the discussion section because we believe that discussing the geographical differences of rural soundscapes is beneficial to the content of related research**(Line 214-257, page 6)**. Secondly, we have improved and supplemented paragraph 3.1 through existing theories**(Line 696-719, page 22)**.

**13. From Line 343 to Line 348, and from Line 386 to Line 392, it is suggested to use the literatures as the theoretical supports for research hypothesis.**

By following the reviewer’s suggestion,we have modified the above two paragraphs to add theoretical support and **the requested changes are presented in bold font** for your quick reference.

**①The problem from Line 343 to Line 348：**The basic view of modern cognitive psychology is that human beings are transmitters of information and systems of information processing. One can categorize things into auditory (sound code), visual (shape code), and semantic (perceptual code) based on different characteristics, and it is believed that au-ditory (sound code) can be used to retrieve previously formed memories through sound . **Hall et al. (2013) found that characteristic sounds rich in historical, cultural, and regional geographic attributes evoke individual sub-jective experiences, past historical memories, and other socio-cultural factors for those who know and are familiar with these sounds. Therefore, sounds not only carry people's memories, but also help people to reproduce their memories, and nostalgia is closely related to reminiscence, which is the reproduction of past life and memories in a positive way. Therefore, those good memories awakened by sound tend to trigger people's nostalgia. It has been shown that soundscape affects human nostalgia, and the sound stimulation received by the senses causes people to feel nostalgic. In the process of rural tourism, attractive soundscape has a positive promotion effect on tourism nostalgia .**Thus, it can be concluded that there is a positive role relationship between rural soundscape perception and nostalgia. （**Line 271-281, page 6**）

**② The problem from Line 386 to Line 392：**Soundscape can directly produce emotional experience . **The theory of place holds that place is a space with special meaning for tourists, and when tourists produce a good tourism experience in this place, they will produce psychological connections such as a sense of dependence and identity [98], and perception also serves an important role in the process of fostering feelings of attachment [99]. Then, travelers may develop place attachment emotions after getting a good Rural Soundscape Perception experience in a rural tourist place. It has been confirmed that soundscape creates a sense of cultural identity and attachment to a place [100].Bartos (2013) et al. [101] used ethnographic meth-ods to investigate and found that children and adolescents formed place at-tachment feelings under the influence of soundscape; Zhang Jie (2018) et al. concluded by analyzing the impact of soundscape on tourism social psy-chology that soundscape perception enhances tourism satisfaction and also strengthens travelers' place attachment, allowing them to identify with the des-tination; Fang Shumiao (2022) et al found that rural sound as a perceived value of rural tourism positively affects place attachment. In addition, Xu Hong (2020) et al[104]found that the perception of odor landscape in rural tourism helps tourists to generate place attachment emotion, and the tourist's tourism process is always carried out in a certain sound environment, sound symbols, together with other sensory symbols such as visual symbols and olfactory symbols, constitute the complete experience of the tourist , so the sound landscape also has a certain place attachment emotion influence.** Based on the above, this research concludes that the perception of rural soundscapes pro-motes place attachment.（**Line 324-346, page 7**）

**14. From Line 376 to Line 383, there is little explanation or theoretical hypothesis for the relationships between the elements in paragraph of 3.2, and the concepts of the words were unclear. It is suggested to modify.**

We really appreciate Reviewer #2's valuable comments and we have systematized paragraph 3.2 by further elaborating the explanation of the relationship between the various elements on the one hand, and the concept of the relevant terms on the other hand.（**Line 266-313, page 6-7**）

**15. What was the aim of paragraph 5.5 Results of Hypotheses Testing? If it is a duplicate of the previous results, it is suggested to delete it. If it is addressing a specific research question, it is recommended to describe the results.**

The purpose of paragraph 5.5, "Results of Hypotheses Testing", is to succinctly summarize all of the above hypothesis results. As stated by reviewer 2, this panel is repetitive with the previous results. In order to simplify the textual content, we have deleted it as suggested by the reviewers.

**16. Although it seems that the basic composition of the questionnaire can be implied in the paper, it is still suggested that the composition of the questionnaire be clearly presented. For example, what are the basic information of the respondents, how to ask about the rural tourism experience, and how to divide the education level.**

We are grateful for this suggestion, and we further propose the composition of the questionnaire by adding the table 2 “Descriptive statistical results” in paragraph of 3.3 Data collection.**（Page 15）**

**17. In the manuscript, H3a of research hypothesis “Personal nostalgia has a significant positive effect on environmental restoration perception” was not valid. It is suggested that this point could be further discussed.**

We are grateful for this suggestion and we have made the following changes in the paper:

**About H3a：historical nostalgia significantly and positively affects Environmental Restoration Perception, while personal nostalgia does not have a significant effect on Environmental Restoration Perception.**

**This result can be explained by several reasons. Firstly，This research reflects on the definition of tourism nostalgia:** personal nostalgia consists mainly of events actually experienced by individuals in the past. Some researchers have argued that nostalgia is a negative emotion involving sadness and pain in an individual's past. It is the process of people's remembrance of the good things in the past, and when the good things that have passed away no longer exist or cannot be reproduced, people feel sadness and helplessness, and this nostalgic emotion of feeling deep remembrance for the things that have passed away is more likely to be the motivation for individuals to escape from the daily routine and to seek after the other, and it becomes an effective way for people to escape from modernity . Therefore, personal nostalgia may be more closely related to tourism nostalgia and have a less significant effect on Environmental Restoration Perception, which has been confirmed by previous scholars' studies .Historical nostalgia focuses on the scenes of people's lives in the past, which can be events that happened in history or memories of a specific historical era . Nostalgia has an aesthetic character, and the subject of nostalgia tends to desire and pursue beautiful things. Since historical nostalgia is an individual's imagination of the past that he or she has not experienced, there is a large space for imagination, therefore, people tend to miss the beautiful and happy historical memories, which is a kind of positive nostalgia emotion that helps to alleviate people's mental stress and physical and mental exhaustion, and then promotes restorative nature. **Secondly, thinking from the perspective of social phenomenon:** the content of personal nostalgia requires real experience, so it is highly subjective, i.e., different experiences of individuals lead to different nostalgia produced by individuals . With the acceleration of urbanization, more and more people are moving from the countryside to the cities, and China's urbanization rate has reached 64.72% as of 2021. The accelerated urbanization, on the one hand, makes many people unfamiliar with the countryside and its soundscape, especially contemporary children or young people, because most of them grew up in the city and know more about the countryside from electronic devices and books, and thus may have a focused and biased memory of the countryside. Therefore, then, the lack of authentic countryside experiences leads to unfamiliarity with the countryside, which in turn makes it difficult for tourists to develop personal nostalgia during tourism. It has been confirmed that the more familiar an individual is with the environment he or she is in, the stronger his or her Environmental Restoration Perception is. So when the familiarity of a place is not enough for tourists to produce personal nostalgia, the lower their Environmental Restoration Perception, so the effect of personal nostalgia on Environmental Restoration Perception is not significant. On the other hand, the acceleration of urbanization has also changed the appearance of the traditional countryside, making the artificial and mechanical sounds in the countryside increase, which makes the tranquil countryside soundscape different from the countryside soundscape in people's recollections, which leads to feelings of sadness and helplessness, and this negative emotion is not conducive to promoting restorativeness.**（Line 760-803, page 23-24）**

（2）**Minor comments**

**1. It is suggested to simplify the topic, such as “Impact of rural soundscape on environmental restoration: An empirical study based on the Taohuayuan Scenic Area in Changde, China”.**

We are grateful for this suggestion and We changed the title to "Impact of rural soundscape on environmental restoration: An empirical study based on the Taohuayuan Scenic Area in Changde, China".**（Line 1-3, page 1）**

**2. Line 204, it is suggested to delete “to fill the gap in the literature”. The manuscript focuses on the increase of measurement items in the specific study case, and whether it is universal needs to be further discussed.**

The content of line 204 was originally part of the literature review section. In response to the reviewer's comments, we have combined the literature review with the introduction, and in doing so have deleted the sentence "to fill the gap in the literature".

**3. Line 235, what is RPRS? When it first appears, it is suggested to write the full name. In addition, this scale is used in this manuscript, and it is suggested to explain the reasons to select and describe the scales in detail in methods parts.**

We are grateful for the suggestion. The following changes have been made: first, the full name of the RPRS has been added**（Line 478, page 12）**. In addition, the reasons for choosing the scale have been detailed in the Methods section**（Line 464-502, page 12）**.

**4. In Table 1, “CH4” and “CH5” should be “CH3”and “CH4”. Please check the details.**

We are sorry for this problem, and it has been carefully changed**（page 13）**. Other data has also been checked.

**5. Figure 2, Figure 3 and Figure 4 were not mentioned in the text. Please check the details.**

We deeply apologize for this problem and have made corrections carefully**（Line 443, page 11 ; Line 459, page 11 ; Line 611, page 19）**. In addition, other images and tables have been carefully checked for mention in the paper.

**6. The words do not have to capitalize the first letter, such as “convergent validity”, or “discriminant validity”. Please check the details.**

Following the reviewer’s suggestion, the problem has been modified.

**7. In Table 3, it is suggested to use words phrases instead of numbers.**

Following the reviewer’s suggestion, the section has been modified**（Line519, page 18）**.

**8. Moreover, it is suggested that the manuscript should undergo extensive English editing.**

Thanks very much for your comments. We have further polished our paper. Please see if the revised version meets the standard of English expression.

We tried our best to improve the manuscript and made some changes in the manuscript. And here we list the changes and mark them in red in revised paper. We appreciate for Editors and Reviewer’ warm work earnestly, and hope that the correction will meet with approval. Thank you very much for your comments and suggestions.
